# Unveiling the landscape of robotic surgery: Insights into Indian medical perspectives

**DOI:** 10.6026/973206300221462

**Published:** 2026-03-31

**Authors:** Vinayak J Shenage, Harshal A Chohatkar, Nitin M Baste, Sanjay P Dhangar, Aishwarya Patil

**Affiliations:** 1 Department of General Surgery, SMBT IMSRC, Dhamangaon, Ghoti, Igatpuri, Nashik, Maharashtra, India; 2 Department of Urology, Bharati Hospital and Research Centre, Pune, Maharashtra, India; 3 Department of Surgery, SMBT IMS & RC, Nashik, Maharashtra, India

**Keywords:** Robotic surgery, India, doctors' perceptions, adoption, minimally invasive surgery, healthcare technology

## Abstract

Limited and variable acceptance of robotic surgery among Indian healthcare providers remains a key challenge for its effective clinical integration. Therefore, it is of interest to evaluate doctors' demographics, knowledge of robotic surgery and perceptions regarding adoption, benefits, drawbacks and future trends. Considerable proportions were uncertain about India's readiness for robotic surgery to replace conventional procedures, although 43.2% believed it may replace laparoscopic surgery in the future. Major concerns included high costs and infrastructure requirements, yet 51.1% expected robotic surgery to become more widespread in India within the next 5-10 years.

## Background:

Robotic surgery has grown widely and expanded in almost all fields of surgery as one of the most innovative developments in the field of surgery [1]. Robotic surgery is a minimally invasive procedure with improved visualization, offering benefits such as smaller incisions, fewer postoperative complications; shorter hospital stays and enhanced healing compared to conventional surgery [2]. In the early 1990s, specifically 1994, the Food and Drug Administration (FDA) approved the use of robots in surgery for the first time. Since then, robotic surgery has evolved extensively and become a cornerstone of modern surgical practice worldwide [3]. While conventional laparoscopy remains a preferred form of Minimally Invasive Surgery (MIS), robotic technology has experienced remarkable growth over the past few decades. The number of robotic-assisted procedures performed globally surged from 80,000 in 2007 to 205,000 in 2010 and continues to rise [4]. Recent estimates suggest that over 11 million robotic surgeries have been performed to date. This surge underscores the increasing adoption of robot-assisted surgery (RAS) across diverse healthcare settings [5]. Robots are gradually being integrated into clinical practice to perform complex operations, including minimally invasive and guided non-surgical procedures [6]. Unlike conventional laparoscopy, current robotic systems offer advancements such as three- dimensional views with depth perception, elimination of hand tremors and a wider range of motion [7]. However, despite these technological advancements, there is a concern that surgeons and medical trainees may not be fully aware or up-to- date with these innovations. In India, a rapidly advancing healthcare landscape, the adoption of robotic surgery presents both opportunities and challenges [8]. Factors such as cost, infrastructure requirements and specialize training influence the pace and extent of its adoption among healthcare providers. Understanding the awareness, perception, knowledge and attitudes of Indian doctors towards robotic surgery is crucial for optimizing its integration into clinical practice and ensuring its benefits are maximized for patients [9]. Therefore, it is of interest to describe the current awareness, perception, knowledge and attitudes towards robotic surgery among Indian doctors.

## Methodology:

This web-based cross-sectional study was conducted in the Department of General Surgery at a tertiary care hospital in North Maharashtra, India. The study aimed to assess the awareness, perception, knowledge and attitudes (KAP) of doctors towards robotic surgery. The study included surgeons (General surgeons, Orthopaedic surgeons, Superspecialists, Gynaecologists, ENT surgeons, Ophthalmologists and others) and physicians across various fields that had access to the internet. Undergraduate medical students were excluded from the study. A total of 600 participants were targeted for inclusion in the study. The KAP (Knowledge, Attitudes and Practices) questionnaire utilized in this study on robotic surgery among doctors in India was meticulously developed to ensure robust validity and reliability. Beginning with an extensive literature review, the questionnaire was initially drafted to encompass critical domains such as awareness, perceptions of benefits and concerns, knowledge of procedures, attitudes towards adoption and reported practices related to robotic surgery. Expert consultation involving surgeons specializing in robotic surgery, medical educators and methodologists refined the questionnaire, focusing on content validity to ensure clarity, relevance and completeness in capturing doctors' perspectives. Pilot testing with a sample of doctors outside the main study group further validated the questionnaire, identifying and resolving ambiguities to enhance its readability and precision. The final questionnaire underwent rigorous assessment for internal consistency reliability using Cronbach's alpha coefficient (above 0.70 considered acceptable), confirming that all items within each construct (e.g., knowledge, attitudes) measured the intended concepts reliably. The questionnaire covered demographic information (age, gender, specialty) and the following KAP domains related to robotic surgery: Knowledge (Awareness of robotic surgery and familiarity with robotic systems); Attitudes (Do you feel India is ready for robotic surgery as a replacement for conventional surgeries?, Do you believe robotic surgery will replace laparoscopic surgery in the near future?, Who do you think benefits most from robotic surgery?, Which of the following is the most important benefit of robotic surgery?, What do you perceive as the disadvantages of robotic surgery?, How often do you receive patient inquiries about robotic surgery?); and Practices (Do you believe robotic surgery will become more widespread in India in the next 5-10 years?, What factors do you think will drive the adoption of robotic surgery in India?, What are the main challenges to the wider adoption of robotic surgery in India?, How can India encourage more widespread use of robotic surgery? and Would you be interested in advocating for or promoting the use of robotic surgery in the future?) Doctors were recruited through social networking websites such as Facebook, Twitter and Whatsapp. Password-protected survey links were posted on these platforms for access to the questionnaire. Participation in the survey was voluntary and not compensated. Informed consent was obtained from each participant prior to participation. Participants were given sufficient time to read, comprehend and answer all questions. Once submitted, answers could not be changed. Participants were given one week to voluntarily complete the questionnaire. Those who did not respond within the defined time frame and after reminders were classified as dropouts and excluded from data analysis. Quantitative data analysis was performed using descriptive statistics including frequencies, percentages, means and standard deviations as appropriate. Comparative analyses between specialties and healthcare sectors were conducted using chi-square tests for categorical variables and t-tests for continuous variables. Ethical approval for this study was obtained from the Institutional Ethics Committee of SMBT IMS & RC, Nashik. Participation was voluntary and confidentiality of participant information was strictly maintained throughout the study. No further contact was made with participants who chose not to participate or withdrew from the study.

## Results:

The study included a diverse sample of 609 doctors, with the majority (43.8%) aged between 36-45 years, followed by those aged 25-35 years (36.1%). A smaller proportion of participants were aged 46-55 years (14.8%), 56-65 years (4.8%) and over 65 years (0.5%). In terms of gender distribution, the majority were male (75.0%), while females constituted 25.0% of the sample. Regarding specialty, the largest group comprised physicians (50.7%), followed by general surgeons (14.4%) and superspecialists (13.6%). Other specialties included orthopaedic surgeons (5.6%), gynaecologists (6.2%), ENT surgeons (4.3%), ophthalmologists (2.3%), plastic surgeons (1.5%) and neurosurgeons (1.3%) (Table 1 - see PDF). The study assessed participants' self-rated knowledge about robotic surgery, revealing that a significant proportion of respondents had limited familiarity with this advanced surgical technique. Specifically, 35.6% of doctors rated their knowledge as "Limited," and 32.7% described their understanding as "Very Limited." Only 23.6% of participants considered their knowledge to be "Average," while a small minority, 8.0%, rated their knowledge as "Advanced" (Figure 1 - see PDF). The study explored various perceptions and attitudes toward robotic surgery among doctors in India. When asked if India is ready for robotic surgery as a replacement for conventional surgeries, 28.9% of respondents answered "Yes," while 32.2% responded "No," and 38.9% were uncertain, indicating mixed opinions about the readiness for such a transition. Regarding whether robotic surgery will replace laparoscopic surgery in the near future, 43.2% believed it would, 24.8% did not and 32.0% were unsure. When considering who benefits more from robotic surgery, a significant majority (77.5%) believed that both surgeons and patients benefit equally, while 11.3% felt that surgeons benefit more and 11.2% felt that patients benefit more. The most important perceived benefit of robotic surgery was "Better assistance in performing delicate surgeries" (32.7%), followed by "Surgical accuracy" (28.9%), "Minimally invasive" (12.0%), "Better ergonomics" (11.2%), "Telesurgery/ remote surgery" (10.0%) and "Faster recovery time" (5.1%). The primary disadvantages of robotic surgery identified were "High cost" (68.0%), "Requires specialized infrastructure" (36.1%), "Limited accessibility" (34.3%), "Long learning curve" (26.4%), "Increased dependency on technology" (24.6%), "Equipment maintenance issues" (24.1%), "Need for additional training and certification" (22.7%) and "Potential for technical malfunctions" (20.2%). Regarding patient inquiries about robotic surgery, 13.6% of doctors reported receiving inquiries very often, 58.3% rarely received inquiries and 28.1% never received inquiries (Table 2 - see PDF). Participants' perspectives on the future adoption of robotic surgery in India revealed optimism, with 51.1% believing it will become more widespread in the next 5-10 years, while 12.5% disagreed and 36.5% were unsure.

Factors perceived to drive the adoption of robotic surgery included "Reduction in costs of Robots" (44.3%), "Advances in technology" (37.4%), "Increased availability of robotic surgery equipment" (30.4%) and "Certificate courses / fellowships / superspeciality courses for training" (26.9%). Challenges to wider adoption include "Cost Barriers" (72.2%), "Logistical Challenges in Setting up Robotic Surgery Units" (29.7%) and "Insufficient Insurance Coverage or Reimbursement Policies" (28.1%). To encourage more widespread use, strategies proposed included "Investment in Training and Education" (60.6%), "Subsidies for Equipment" (43.2%) and "Partnerships with Global Leaders in Robotic Surgery" (37.4%). A significant majority (63.2%) of participants expressed interest in advocating for or promoting the use of robotic surgery in the future, highlighting potential support for its integration into mainstream medical practices in India (Table 3 - see PDF). Gender-based differences in perceptions of robotic surgery among Indian doctors were analyzed, revealing significant associations across several variables. While a higher proportion of males demonstrated advanced knowledge (9.4%) compared to females (3.9%), this difference did not reach statistical significance (p = 0.06). More males believed in India's readiness for robotic surgery as a replacement for conventional surgeries (27.8%) compared to females (32.2%) (p = 0.005) and anticipated a future where robotic surgery replaces laparoscopic procedures (43.5% vs. 42.1%, p = 0.0005). Both genders perceived equal benefits for surgeons and patients from robotic surgery (p = 0.92). Males reported more frequent patient inquiries about robotic surgery (15.5%) than females (7.9%) (p = 0.007) and were more optimistic about its widespread adoption in India over the next 5-10 years (52.7% vs. 46.1%, p = 0.018). Moreover, a greater percentage of males expressed interest in advocating for or promoting robotic surgery (66.3% vs. 53.9%, p = 0.02) (Table 4 - see PDF). This study investigated perceptions of robotic surgery among doctors in India across different age groups. Analysis revealed no significant differences in knowledge about robotic surgery across age groups (p = 0.648). Similarly, perceptions on whether India is ready for robotic surgery as a replacement for conventional surgeries did not vary significantly by age (p = 0.352). However, significant differences were observed in beliefs about robotic surgery replacing laparoscopic surgery, with younger age groups (57.7% in <36 years) more optimistic compared to older age groups (25.6% in 46-55 years) (p < 0.0001). Regarding who benefits more from robotic surgery, there were no significant age-related differences (p = 0.633). Frequency of patient inquiries about robotic surgery also showed no significant variation across age groups (p = 0.654). Similarly, beliefs about the future widespread adoption of robotic surgery in India did not differ significantly by age (p = 0.669). Interest in advocating for or promoting robotic surgery in the future also did not vary significantly across age groups (p = 0.356) (Table 5 - see PDF).

## Discussion:

This study aimed to explore the awareness, perceptions, knowledge and attitudes towards robotic surgery among doctors in India, providing insights into their readiness and acceptance of this technology in clinical practice. The findings reveal several noteworthy points that contribute to the broader understanding of robotic surgery adoption in India's medical landscape. The study found varied levels of knowledge about robotic surgery among participants, with a majority reporting limited to average knowledge. This aligns with previous research indicating that while robotic surgery has gained momentum globally, there remains a significant gap in comprehensive understanding among medical professionals [10]. The findings underscore the need for targeted educational interventions to enhance awareness and knowledge dissemination about robotic surgical techniques. Participants exhibited diverse perceptions regarding the readiness of India for robotic surgery as a replacement for conventional procedures. A significant proportion expressed uncertainty (38.9%) about its readiness, while a substantial number (32.2%) remained skeptical. These findings contrast with studies in developed nations where higher acceptance rates and early adoption of robotic surgery have been documented [14]. Factors contributing to this discrepancy in adoption readiness could include cost considerations, infrastructural limitations and varying degrees of exposure to robotic technology. Notably, a considerable percentage (43.2%) of participants believed that robotic surgery would replace laparoscopic surgery in the near future. This perception reflects a shift in surgical preferences towards more advanced, minimally invasive techniques, which promise improved precision and patient outcomes. Similar trends have been observed globally, suggesting a growing confidence in robotic systems' capabilities to surpass traditional laparoscopy [11].

The perceived benefits of robotic surgery primarily centered on enhanced surgical precision (28.9%), minimally invasive procedures (12.0%) and improved ergonomics (11.2%). These advantages align with established literature highlighting robotic systems' ability to mitigate surgeon fatigue, improve dexterity and facilitate complex maneuvers in confined spaces [12]. Conversely, concerns over high costs (68.0%), specialized infrastructure requirements (36.1%) and a steep learning curve (26.4%) were identified as significant barriers to widespread adoption. These findings resonate with global studies emphasizing the economic and logistical challenges associated with integrating robotic surgery into healthcare systems. The frequency of patient inquiries about robotic surgery varied, with a majority of respondents indicating rare patient interest (58.3%). This discrepancy underscores the need for comprehensive patient education and awareness campaigns to bridge the gap between technological advancements and public understanding. Studies from Western countries suggest that increased patient awareness positively correlates with higher acceptance rates and demand for robotic-assisted procedures [13]. Looking ahead, respondents expressed cautious optimism about the future widespread adoption of robotic surgery in India over the next 5-10 years (51.1%). Factors perceived to drive adoption included technological advances (37.4%), reductions in equipment costs (44.3%) and government support (21.8%). Addressing the identified challenges such as cost barriers, regulatory hurdles and limited training programs will be crucial in fostering a conductive environment for robotic surgery integration. Strategies like investment in training, subsidies for equipment and strategic partnerships with global leaders emerge as pivotal steps towards enhancing accessibility and affordability [14].

## Conclusion:

This study reveals varied levels of knowledge and perceptions among doctors in India regarding robotic surgery, with opportunities and challenges identified. Despite cautious optimism about its potential, barriers such as high costs, infrastructure needs and the requirement for specialized training persist. To address these issues, targeted educational initiatives, strategic investments in technology and supportive policies are crucial for the effective integration of robotic surgery into India's healthcare system.

## Advancement to knowledge:

This study provides evidence-based insight into clinicians' perspectives, highlighting priority areas for training, policy planning and cost-related interventions to support robotic surgery adoption in India.
